# Deucravacitinib versus placebo in adults with moderate-to-severe hidradenitis suppurativa: A pilot study

**DOI:** 10.1016/j.jdin.2026.08.009

**Published:** 2026-09-03

**Authors:** Martina L. Porter, Maneli Doroudian Tehrani, Nazrin Ashina, Bruna G.O. Wafae, Thao-Mary Tran, Hoang Truong, William Sweet, Julia L. Gao, Prerna Salian, Alexa B. Kimball

**Affiliations:** a Clinical Laboratory for Epidemiology and Applied Research in Skin (CLEARS), Department of Dermatology, Beth Israel Deaconess Medical Center, Boston, Massachusetts; b Harvard Medical School, Boston, Massachusetts; c Department of Medicine, Santa Barbara Cottage Hospital, Santa Barbara, California

**Keywords:** clinical efficacy endpoint, clinical trial, deucravacitinib, hidradenitis suppurativa, patient-reported outcome, TYK2 inhibitor

*To the Editor:* Hidradenitis suppurativa (HS) is a disabling inflammatory skin disease characterized by recurrent painful nodules, abscesses, and fistulas that impair quality of life.^1^ Despite the expansion of biologic therapies, many patients remain refractory to available treatments. JAK inhibitor trials have shown promise in moderate-to-severe HS which support the JAK/STAT pathway as a potential therapeutic target.^2^^,^^3^ Tyrosine kinase 2, an intracellular enzyme within the JAK-STAT pathway, mediates signaling through IL-12, IL-23, and type I interferons, and contributes to NK cell function, B-cell development, and Th1/Th17 differentiation. Deucravacitinib is an oral, selective tyrosine kinase 2 inhibitor that binds to the pseudokinase domain.^4^

We conducted a pilot study evaluating deucravacitinib in moderate-to-severe HS. This single-center, randomized, double-blind, placebo-controlled pilot study (NCT05997277) enrolled adults, with moderate-to-severe HS, defined by the presence of at least 5 abscesses and inflammatory nodules (ANs) involving at least 2 distinct anatomical regions. Participants with more than 20 draining fistulas at baseline, or recent initiation or use of biologic or non-biologic HS therapies within protocol-defined washout periods were excluded. Participants were randomized 2:1 to receive deucravacitinib 6 mg orally twice daily or placebo for 16 weeks. The primary endpoint was change in AN count at week 16. Secondary outcomes included Hidradenitis Suppurativa Clinical Response, Dermatology Life Quality Index, pain numerical rating scale, and safety assessments. Due to a change in funding availability, the study was terminated early, and the resulting analyses are exploratory in nature.

Thirteen participants were screened and 7 randomized, including 5 assigned to deucravacitinib and 2 to placebo (Fig 1). Most had Hurley stage II disease, with a mean baseline AN count of 7.43 (SD 4.37). Adverse events were reported in 6 participants with no serious adverse events and consisted of upper respiratory infections, gastrointestinal symptoms, and headaches, all resolving without treatment discontinuation. One participant from the deucravacitinib-treated group received traimcinolone injections after developing two draining fistulas, which resolved completely by week 16. All randomized participants were included in the population used for baseline characteristics, safety, and efficacy analyses. Missing data were imputed using last observation carried forward. Median AN counts decreased from baseline to week 16 in both groups, with a median paired reduction of 2 ANs (IQR 2-4) in the deucravacitinib and 9 ANs (IQR 4-14) in the placebo group. Although 4 out of 5 in deucravacitinib group achieved Hidradenitis Suppurativa Clinical Response at week 4, this response was not sustained through week 16 (Fig 2). Mean Dermatology Life Quality Index improvement was −4.6 (SD 5.73) in the treatment arm versus −2.0 (SD 1.41) with placebo, and 60% of deucravacitinib-treated participants achieved a clinically meaningful Dermatology Life Quality Index reduction of at least 4 points, compared with none in the placebo arm. Three of 5 participants in the deucravacitinib group had reduced pain numerical rating scale scores, 1 was unchanged, and 1 worsened; both placebo participants had higher pain numerical rating scale scores at week 16 than at baseline.

**Fig 1:**
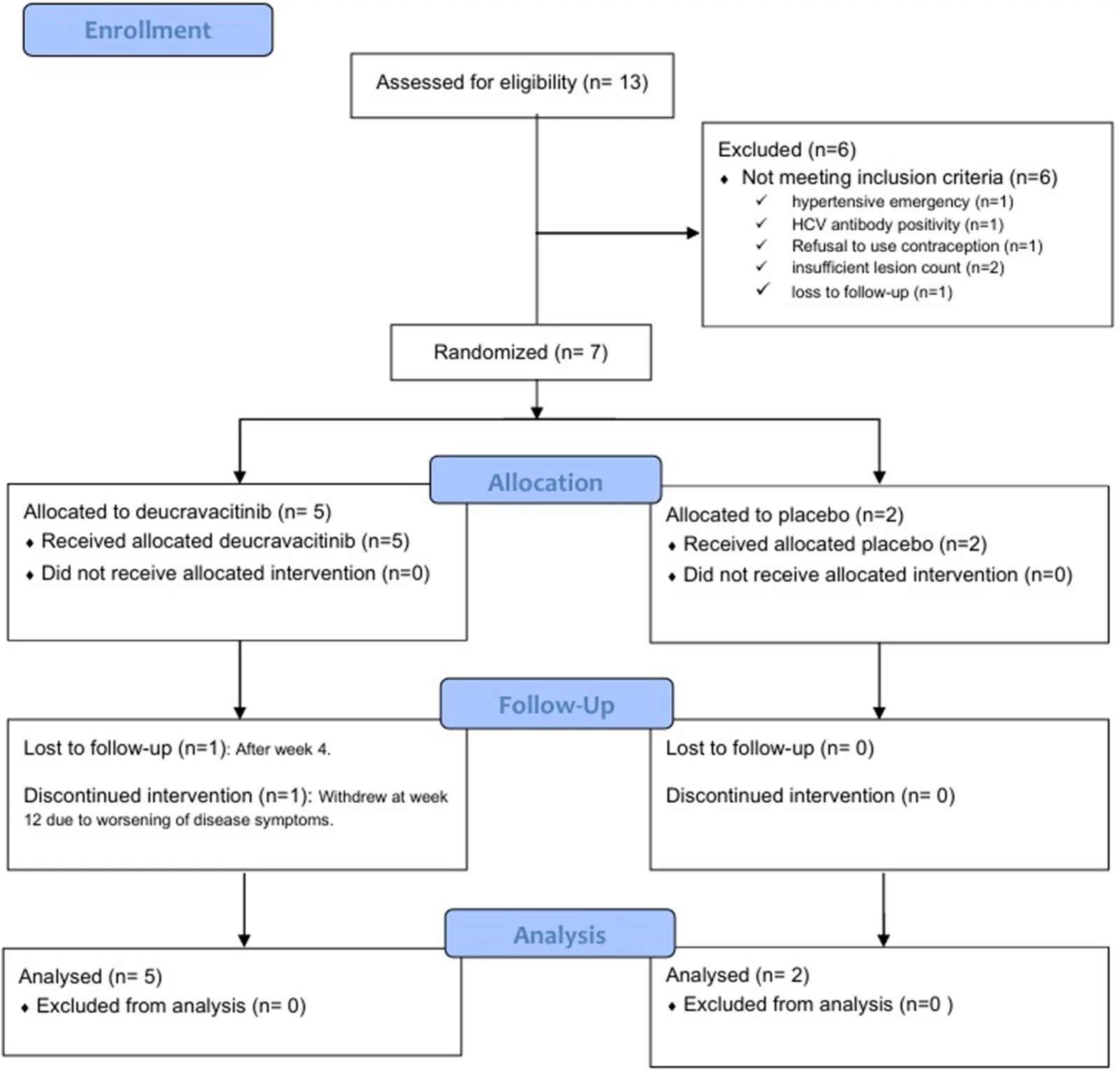
Hidradenitis suppurativa. CONSORT flow diagram of participant enrollment, randomization, allocation, follow-up, and analysis.

**Fig 2:**
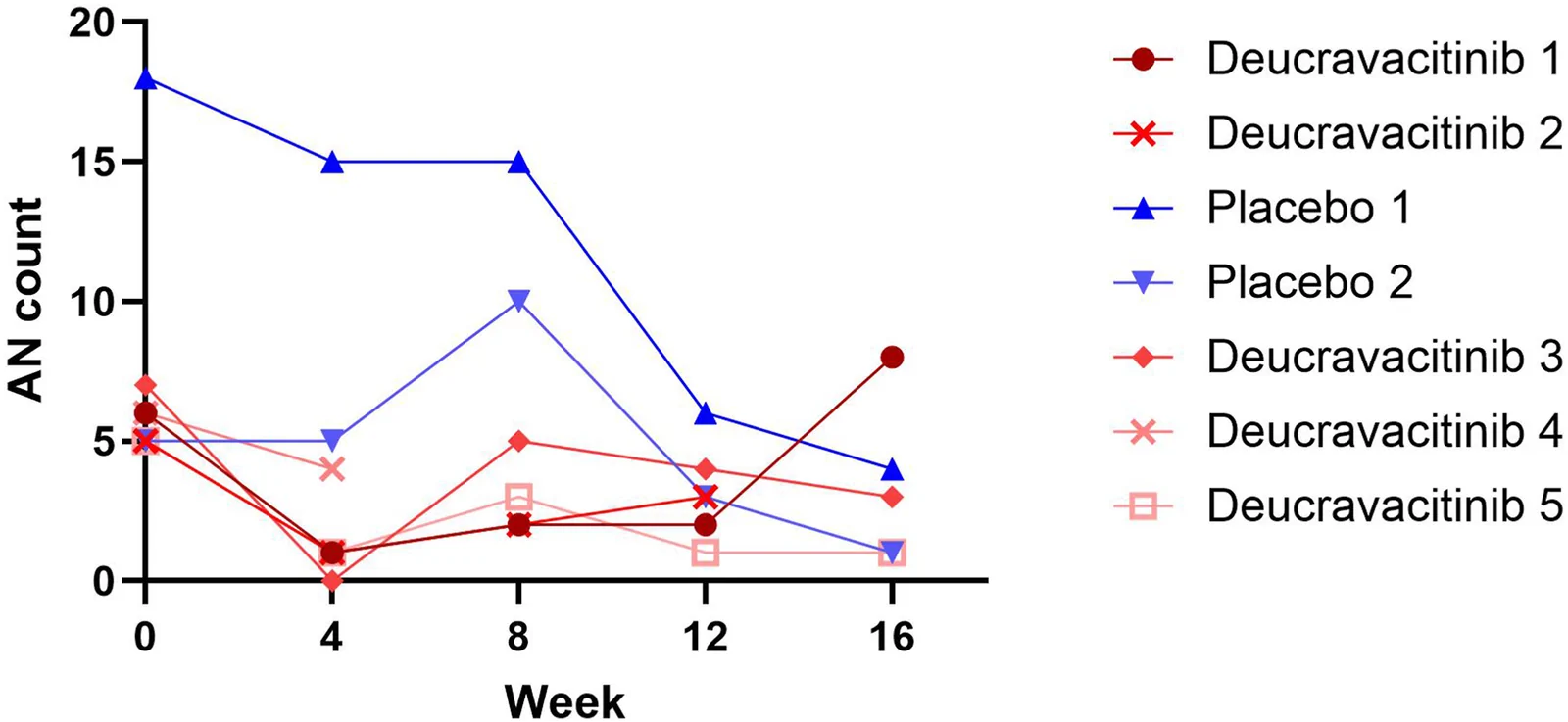
Hidradenitis suppurativa. Individual trajectories of abscess and inflammatory nodule (AN) counts from baseline through week 16 in participants randomized to deucravacitinib (*red lines*) or placebo (*blue lines*). Each *line* represents 1 participant, and data shown are observed values only. Participants who discontinued the study are marked with an X. No imputed values are displayed in the figure. Although missing data were handled using the last observation carried forward (LOCF) method for the intention-to-treat efficacy analyses, only observed values are presented in the plotted trajectories. *AN*, Abscess and inflammatory nodule.

The limited sample size, premature study termination, fluctuating course of HS, and high placebo response prevent definitive conclusions.^5^ While no pattern of differences could be detected, this pilot study was underpowered to evaluate efficacy or safety.

## Conflicts of interest

Authors Sweet, Tran and Dr Ashina are study coordinators for studies sponsored by AbbVie, Avalo, Bayer, Bristol Myers Squibb, Eli Lilly, Incyte, MoonLake, Immunotherapeutics AG, Novartis, Oasis Pharmaceuticals, Pfizer, Prometheus Laboratories, Regeneron, Sonoma Biotherapeutics, Sanofi, and UCB. Drs Doroudian Tehrani, Gao, and Wafae are subinvestigators for AbbVie, Avalo, Arcutis, Takeda, Zurabio, Eli Lilly, Incyte, MoonLake, Merck, Prometheus, Sanofi, Insmed, UCB, Bristol Myers Squibb, and Novartis. Dr Gao has received fellowship funding from Janssen and AbbVie. Dr Porter is a consultant for and received grants from AbbVie, Avalo Therapeutics, Arcutis, Aristea Therapeutics, Eli Lilly and Company, Incyte, Janssen, Merck, MoonLake Immunotherapeutics, Navigator Biosciences, Novartis, Pfizer, Prometheus, Sanofi, Sonoma Biotherapeutics, UCB, Ventyx, Zura Bio; consultant for Almirall, Citryll, FIDE, Insmed, Oruka, Otsuka, Propeller Biosciences, Spyre, Trifecta Clinical/WCG; received grants from AnaptysBio, Bayer, Bristol Myers Squibb, OASIS Pharmaceuticals, and Regeneron; received royalties from Beth Israel Deaconess Medical Center; and received fellowship funding to institution from AbbVie. Dr Kimball received grants from AbbVie, Acelyrn, AnapytsBio, Avalo, Bristol Myers Squibb, Eli Lilly, Incyte, Insmed, Janssen, MoonLake, Novartis, Pfizer, Prometheus, Sanofi, and Sonoma Bio; received consulting fees from AbbVie, Avalo, Boehringer Ingelheim, Cellarity, Citryll, Curogen, Eli Lilly, Evoimmune, Janssen, Merck, MoonLake, Incyte, Novartis, Nurix, Pfizer, Sanofi, Regeneron, Takeda, Target RWE, UCB, Union Therapeutics, Ventyx, and Zura Bio. They also served on the board of directors of Almirall. Authors Truong and Salian have no conflicts of interest to declare.
